# How can Schools of Public Health Actively Promote Peace?

**DOI:** 10.3389/phrs.2021.1604459

**Published:** 2021-10-11

**Authors:** Yudit Namer, Lisa Wandschneider, John Middleton, Nadav Davidovitch, Oliver Razum

**Affiliations:** ^1^ Department of Epidemiology and International Public Health, School of Public Health, Bielefeld University, Bielefeld, Germany; ^2^ The Association of Schools of Public Health in the European Region (ASPHER), Brussels, Belgium; ^3^ School of Public Health, Ben-Gurion University of the Negev, Be’er Sheva, Israel

**Keywords:** schools of public health, peace, conflict, war, social justice, equity

War has detrimental consequences on public health. They range from medical and public health implications, human rights violations, and ethical, political, social, environmental, and economic implications [1]. With such profound influence on health, Schools of Public Health (SPH), intrinsically, will oppose organized armed conflict. But can they do more? We believe so. SPH can–and should–actively engage in promoting peace. They should do so not only in terms of the absence of war or conflict but also to promote “the presence of justice and equity, as well as respect for human rights and for the Earth” [2].

Medical doctors, nurses, and other health professions have already been active in this, e.g., the International Physicians for the Prevention of Nuclear War (IPPNW). Also, the framework Peace through Health (PtH) [3] promotes the vision to collaborate “on policy development, training, and service across borders and lines in conflict” [4]. The PtH approach, however, primarily addresses the role of health and humanitarian aid workers in zones of armed conflict and those involved in rebuilding afterward. Such workers are without doubt of utmost importance for immediate peacebuilding processes. Many of these approaches are primarily medical or humanitarian. Public health, with its interdisciplinary perspective rooted in the understanding of the role of social, economic, and political determinants of health as well as on prevention and population approaches, has a lot more to offer [5]. It can broaden the discourse and practices on how to engage in conflicts and war prevention.

## What role is there for Schools of Public Health?

SPH should play a more active role in promoting peace for three reasons. First, bringing in SPH as the main places for training and research has the potential to broaden the frame and scope to reflect how public health players from many disciplines contribute to promoting peace, e.g., social epidemiology, health statistics, public health law and ethics, health politics, environmental health, and One Health.

Second, SPH have a strong commitment to practice as well as to theory to protect population health in its broadest definition. Thus, SPH bring multiple levels and sectors together, from grassroots to policymakers [6].

Third, SPH have the tools to provide evidence. For example, they can explore the mental as well as physical health consequences of armed conflict and other political determinants of health over the life course expressed at different levels from individual to community [6].

Hence, there are theoretical and practical connections between public health and peacebuilding: both pay attention to factors intersecting at the individual, community, societal, and environmental level, e.g., through a socioecological, system-orientated approach. In addition, both incorporate a social justice perspective, focusing on resources and resilience as well as critical view on power hierarchies [7]. These shared objectives provide rich opportunities for transdisciplinary collaboration. In other words, “health promotion is peace promotion” [8].

## What actions can Schools of Public Health take?

We suggest building peace through SPH by following selected aspects of Santa Barbara and MacQueen's “peace through health mechanisms” [3]:

*Use of health-related superordinate goals*: SPH should be active in producing critical research on 1) health consequences of conflict and health-based theorization of peace that incorporates layers from the interpersonal to international, insisting on wellbeing/flourishing rather than absence of ill-health from the individual to the environment, and 2) the understanding of inequities as root causes of conflict and ill-health, thereby acting to prevent both.

*Discovery and dissemination of facts*: SPH should participate in research practices that contextualize conflicts (e.g., within the frame of colonialization) in cooperation with communities affected by conflict and actively disseminate knowledge that could inform peacebuilding activities.

*Diplomacy, mediation, and conflict transformation*: SPH should play active intermediary and transformative roles by creating spaces for students and others on different sides of divides in a way that takes into account mechanisms and asymmetries of power and principles of social justice.

*Solidarity and support*: SPH, in their educative capacity, should actively try to instill the relational skill of mutuality and recognition in students which would facilitate active refusal of dehumanization. SPH should stand in solidarity with communities affected by conflict by creating spaces for people surviving oppression (e.g., hosting scholars/students at risk) and being vocal witnesses to human rights violations inherent in conflict.

*Dissent and non-cooperation*: SPH should actively and vocally refuse to participate in activities that go against peacebuilding. They should actively stand against academic organizations which are affiliated with conflict and rights violations.

Guided by these mechanisms, SPH will become active agents in promoting healthy and peaceful societies based on the principles of social justice and equity. Challenges such as the COVID-19 pandemic and climate change show the need for regional and global collaboration [9]. Public health, deeply embedded in social and political roots, has the potential of broadening our perceptions of solidarity. This is not an easy journey, but one we must embark on.
