# Corrigendum: How Can Schools of Public Health Actively Promote Peace?

**DOI:** 10.3389/phrs.2022.1605297

**Published:** 2022-09-29

**Authors:** Yudit Namer, Lisa Wandschneider, John Middleton, Nadav Davidovitch, Oliver Razum

**Affiliations:** ^1^ Department of Epidemiology and International Public Health, School of Public Health, Bielefeld University, Bielefeld, Germany; ^2^ The Association of Schools of Public Health in the European Region (ASPHER), Brussels, Belgium; ^3^ School of Public Health, Ben-Gurion University of the Negev, Be’er Sheva, Israel

**Keywords:** schools of public health, peace, conflict, war, social justice, equity

In the published article, there was an error.

In the original article, the citation for “**Santa Barbara and MacQueen**” was incorrectly written as “**Santa Barbara and Queen**”. It should be “**Santa Barbara and MacQueen**”.

A correction has been made to **What actions can Schools of Public Health take?** Paragraph 1. This sentence previously stated:

“We suggest building peace through SPH by following selected aspects of Santa Barbara and Queen’s “peace through health mechanisms” [3]:”

The corrected sentence appears below:

“We suggest building peace through SPH by following selected aspects of Santa Barbara and MacQueen’s “peace through health mechanisms” [3]:”

The authors apologize for this error and state that this does not change the scientific conclusions of the article in any way. The original article has been updated.

